# *Aristolochia clematitis* L. Ethanolic Extracts: In Vitro Evaluation of Antioxidant Activity and Cytotoxicity on Caco-2 Cell Line

**DOI:** 10.3390/plants13212987

**Published:** 2024-10-25

**Authors:** Maria-Alexandra Pricop, Alexandra Teodora Lukinich-Gruia, Iustina-Mirabela Cristea, Virgil Păunescu, Călin Adrian Tatu

**Affiliations:** 1 OncoGen Centre, County Hospital Pius Branzeu, 156 Liviu Rebreanu Blvd., 300736 Timisoara, Romania; alexandra.pricop@oncogen.ro (M.-A.P.); mirabela.cristea@oncogen.ro (I.-M.C.); vpaunescu@umft.ro (V.P.); 2 Department of Applied Chemistry and Environmental Engeneering and Inorganic Compounds, Faculty of Industrial Chemistry, Biotechnology and Environmental Engeneering, Polytechnic University of Timisoara, Vasile Pârvan 6, 300223 Timisoara, Romania; 3 Department of Functional Sciences, Center of Immuno-Physiology (CIFBIOTEH), University of Medicine and Pharmacy “Victor Babes”, Eftimie Murgu Sq. 2, 300041 Timisoara, Romania

**Keywords:** *Aristolochia* sp. plants, antioxidant capacity, ethanolic leaves extract, colon cancer cell line

## Abstract

*Aristolochia* sp. plants are used in traditional medicine because of their immunostimulatory and anticarcinogenic properties, despite their content of aristolochic acids (AAs), carcinogenic and nephrotoxic agents. Therefore, ethanolic extracts of *Aristolochia clematitis* leaves, a specie growing in Western Romania, were obtained to study antioxidant and cytotoxic effects. The antioxidant capacity of the extract was evaluated by five in vitro chemical-based assays, proving that ABTS assay was a better method for this type of evaluation showing an IC_50_ of 160.89 ± 0.21 µg/mL. Furthermore, the cytotoxic effects of the extract were established by an IC_50_ of 216 µg/mL for 24 h by MTT assay, followed by a cell-based assay on Caco-2 cells by the ABTS method. The antioxidant effects of the *A. clematitis* extract demonstrate potential therapeutic applications in complementary medicine.

## 1. Introduction

Medicinal plants were the first types of treatment for health conditions since ancient times for a wide spectrum of diseases and medical conditions. Recognizing the medicinal significance of indigenous plants, the World Health Organization (WHO), in its 1997 guideline, stated that “effective locally available plants can be used as substitutes for drugs” [1]. Nowadays, almost 50% of new drugs have been patterned after natural phytochemical compounds [1]. Interest in the development of innovative natural remedies based on medicinal plants to protect human health has increased, especially in developing countries, by over 80% during the last decades [2,3].

In Europe, Asia, and South America, *Aristolochia* spp. has been utilized as herbal medicine for more than 2000 years. *Aristolochia* sp. plants are useful in treating a wide range of conditions, as they have anti-inflammatory, antioxidant, antimicrobial and immunostimulatory properties [4,5], as well as medicinal effects that have been thoroughly demonstrated to be due to a wide array of bioactive compounds [6]. On the other hand, these plants were forbidden to be used due to their content in aristolochic acids (AAs), nephrotoxic, carcinogenic, and mutagenic compounds [7,8]. The short-term exposure (up to one year) to AAs lead to aristolochic acid nephropathy (AAN) [9,10,11,12,13], and a longer term exposure (up to several decades) can lead to Balkan endemic nephropathy (BEN) [14,15]. Both of these kidney diseases are associated with urothelial carcinoma [16], and with similar clinical expression and pathological lesions [16].

Even if these plants were forbidden by the US Food and Drug Administration and regulatory authorities of some countries [17], it is still used in the form of drugs or phytomedical preparations [7,18], especially in Asia [1], due to the beneficial effects of their short-term consumption [2,19,20,21]. The beneficial effects of bioactive molecules are attributed to secondary compounds such as alkaloids, terpenoids and polyphenolic compounds [7,8,22], these compounds being powerful antioxidants that fight free radicals, which result from respiration and metabolic processes [20]. Free radicals resulting from AAs’ effects are reactive and hence, oxidative molecules and their overproduction can lead to oxidative stress, which is linked to inflammation, kidney disease, and is associated with cancers [7,8,23].

An understanding of the biological properties of *Aristolochia clematitis*, the most frequent species found in Europe [7,18,23], can facilitate the identification of compounds with therapeutic potential. The *Aristolochia* species were already screened for their chemical composition; therefore, secondary metabolites, classified according to chemical structure, including aristolochic acids and esters, aristolactams, aporphines, protoberberines, isoquinolines, benzylisoquinolines, amides, flavonoids, lignans, biphenyl ethers, coumarins, tetralones, terpenoids, benzenoids, and steroids have been thoroughly characterized [5,24,25,26]. Other classes of compounds were identified by gas-chromatography analyses from *A. tagala* leaves extracted in different types of solvents, hydroalcoholic and methanolic, showing the most diverse biologically potent compounds (e.g., alkaloids, flavonoids) [27]. Also, *A. clematitis* from Romania, prepared as tincture and infusion from roots, steams, leaves was analyzed by two types of gas-chromatography revealing various classes of compounds, from fatty acids to terpenic compounds, coumarins and alkaloids, and aristolochic acids derivatives, all with therapeutic activities [28]. Another study on *A. clematitis*, but from Algeria, showed that organic solvents and water extracts from roots possess similar composition (e.g., terpenoids, alkaloids, fatty acids) [29]. These compounds could aid in developing new drugs with antioxidant capabilities, potentially treating various conditions linked to oxidative stress [30], while encouraging the sustainable exploitation of plant resources [31]. The extraction process to obtain bioactive substances are preferentially carried out using ethanol as an extraction solvent, given its high affinity for both lipophilic and hydrophilic bioactive molecules. Also, ethanol is the most commonly used solvent because of its economical and reusability value as well as low toxicity [32]. Limited studies on ethanolic extract from *A. clematitis* aerial parts demonstrated through several chemical-based antioxidant assays, that the extract had good antioxidant activity with polyphenolic and flavonoids content methods [33]. Chemical-based assays are preferred to be used in an initial screening process of the natural products because they are low cost, simple and rapid procedures, with cheap reagents, and require simple instrumentation (e.g., spectrophotometer) [34,35].

The major pathway for herbal products containing AAs to the kidneys, where the effects are irreversible by long-term exposure, is through the digestive system, after absorption and passage through the intestinal cells; these cells are vulnerable to oxidative damage due to the exposure to luminal oxidants and toxins from ingested foods [36,37]. Medicinal plants are used in the treatment of colon cancers [38], their effects being investigated on various human cell lines derived from the intestinal mucosa. Caco-2 is such a cell line derived from a human colorectal adenocarcinoma, and represents a reliable proxy model of intestinal absorption of AAI [37,38].

**Figure 1:**
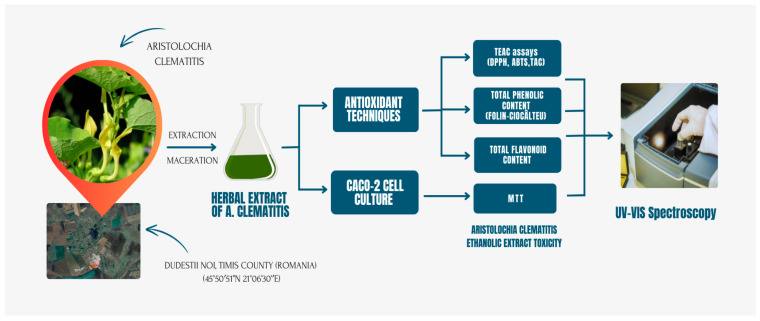
A synoptic view of the experiments performed.

Our study aimed to investigate in more detail the in vitro antioxidant properties and in vitro cytotoxic activity of the *Aristolochia clematitis* extract. As such, we used dried leaves as biological material and had them extracted at room temperature by maceration with ethanol, which has a similar formulation with the one used in ethnomedicine. The extract was used for the evaluation of antioxidant activities with five different chemical assays. Four out of the five assays were chosen to compare our extract with the literature data: 1,1-diphenyl-2-picrylhydrazyl radical (DPPH), total antioxidant capacity (TAC), total flavonoids content (TFC) and total polyphenolic content (TPC). The ABTS assay was chosen as a different method; to our knowledge this method was used for the first time in the literature to test *Aristolochia* sp. extracts or compounds for antioxidant activity. Also, the ABTS method can be used both as a chemical based-assay and cell-based assay. Before testing the extract’s antioxidant activity on the Caco-2 cell line, the extract’s toxicity was evaluated to establish an inhibitory effect. After the concentration of the extract, which inhibited 50% of the cells, was established, an antioxidant effect by the ABTS assay was evaluated. The sequence of the experimental design is depicted in Figure 1.

## 2. Results

### 2.1. The Antioxidant Activities of A. clematitis Leaf Ethanolic Extract

To assess the antioxidant potential of *A. clematitis* leaf extracts, five distinct assays were employed, and three of them used Trolox as standard; the findings delineating the antioxidant capacity are detailed in Table 1. Antioxidants are able to reduce the color intensity of DPPH, ABTS, and phosphomolybdate solutions due to their ability to donate protons; these effects have been quantified using as standard, the Trolox equivalent antioxidant capacity. The IC_50_ values for DPPH and ABTS were calculated by graphing concentrations of the extract against the proportional antioxidant inhibition percent [39,40]. Another Trolox method used was the total antioxidant capacity (TAC) using the phosphomolybdate reagent [41,42]. The other two methods were based on propyl gallate and quercetin calibration curves and the spectrophotometric measurement of the total polyphenolic content and the total flavonoid content. Results are obtained from three independent experiments, with each sample plated in quadruplicate; these are presented in Table 1.

Comparing the DPPH and ABTS results, the first method gave values almost three times higher than the ABTS ones. The extract sample values were similar to those of Trolox for each of the methods. The TAC method gave values almost 10 times higher than DPPH and 25 times higher than ABTS. An IC_50_ value more than 150 µg/mL represents weak antioxidant activity [43]. This indicates that the ABTS method could be a better method to demonstrate the antioxidant activity of the ethanolic extract. The total phenolic content has a low value, showing a low content in polyphenols, but the total flavonoid content has a high activity suggesting a high content in flavonoids. Therefore, a correlation between TPC and TFC assays shows that the method of extraction with ethanol at room temperature is a proper method for flavonoids extraction.

### 2.2. Evaluation of Cytotoxicity of Ethanolic Extract on Caco-2 Cells

**Figure 2:**
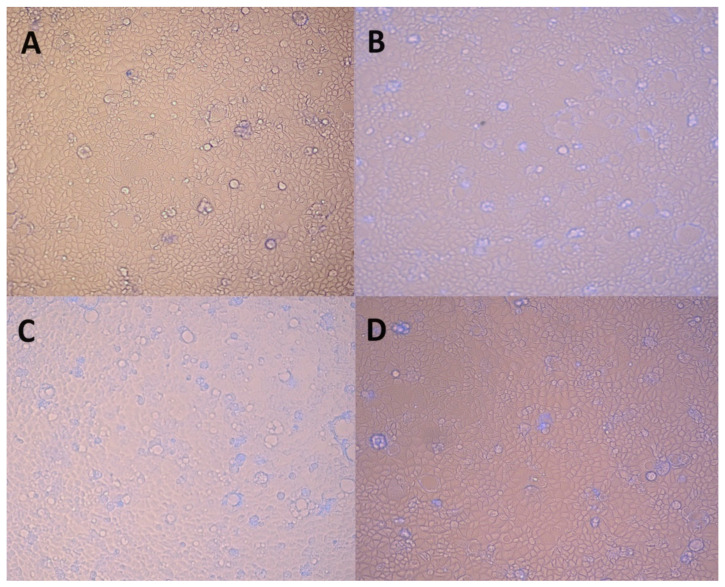
Morphological assessment of Caco-2 cells (control vs. treatment after 24 h of incubation) exposed to (**A**). AcE (150 µM), (**B**). Trolox (2 mM), (**C**). ethanol, and (**D**). control cells.

The effects of Trolox and extracts on Caco-2 cells were evaluated by the MTT assay. Five dilutions of *A. clematitis* ethanolic extracts of 100, 200, 300, 500, and 600 µg/mL were used to obtain an IC_50_ for both 24 and 48 h. The same procedure was used for the calculation of the IC_50_ of Trolox for the three different concentrations ranging from 0.5 to 0.125 mg/mL for 24 h. The results obtained by the MTT assay for 24 h and 48 h were 216 µg/mL and 224 µg/mL, respectively, and 207 µg/mL for Trolox. The cells were photographed for morphological changes at 100x magnification (Figure 2).

The Caco-2 cells did not show any morphological differences upon exposure to the *Aristolochia* extract or Trolox, with the effect being similar (Figure 2C,D). These results suggest that the short-term exposure of Caco-2 cells to the extract does not affect their morphology, at least at the tested concentration.

### 2.3. Evaluation of Antioxidant Capacity on Caco-2 Cells by ABTS Assay

**Figure 3:**
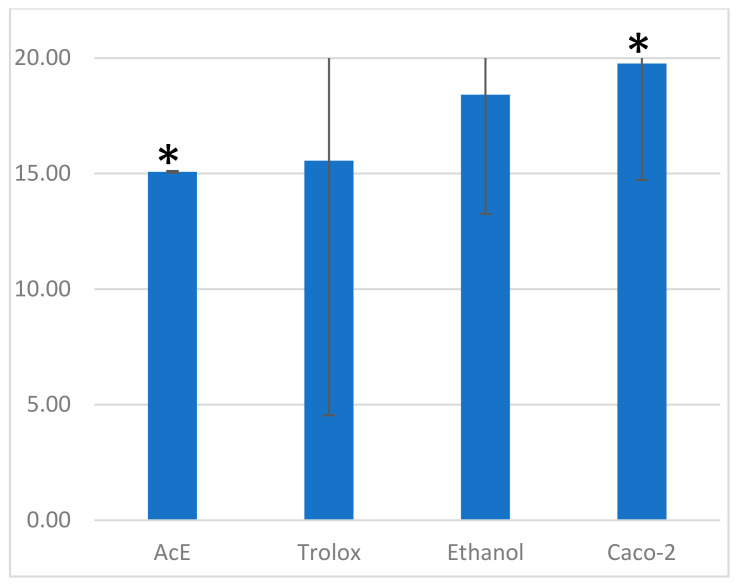
Graphical representation of the ABTS results obtained on Caco-2 cells treated with *A. clematitis* ethanolic extract (AcE, 150 µM); Trolox (2 mM); ethanol as the solvent control; and Caco-2 absolute control cells. *—samples statistically different (*p* < 0.05).

Antioxidant activity with the ABTS method was performed after establishing an IC_50_ value and the chosen concentration for the ABTS assay was 150 µM for ethanolic extract, and 2 mM for Trolox. The antioxidant activity in Caco-2 cells was assessed by the ABTS assay [40]. Antioxidant activity was expressed as mg Trolox Equivalents (TE)/10^6^ cells. Results obtained by the ABTS method were compared (Figure 3).

Ethanolic extract-treated cells had an antioxidant activity closer to Trolox, and control cells had closer results to ethanol treated ones. Samples were compared by single factor ANOVA statistical analysis and only cells treated with the extract exhibited statistically significant antioxidant activity compared to the control group (*p* < 0.05).

## 3. Discussion

Human health is affected by oxidative stress, which appears as an imbalance between oxidants and antioxidants in favor of oxidants [44]. Regarding the fact that in the present study a natural product was evaluated, chemical and cell-based assays were used. Through these methods, different aspects of the compounds reactivity toward ROS was evaluated [45]. This study contributes to the literature with new data on *A. clematitis* ethanolic extracts and their analysis on some new antioxidant assays. In the scientific literature, the antioxidant assays employed were total phenolic content, flavonoid content, reducing power, ferrous metal ions chelating, superoxide anion, hydroxyl radical, hydrogen peroxide, and nitric oxide [33,46]. Based on Lerma et al. (2022), other species of *Aristolochia* extracts were evaluated for their antioxidant and anticancer activity [23].

*A. clematitis* ethanolic extracts demonstrated promising DPPH radical scavenging activity with an IC_50_ value of 391.62 ± 0.06 μg/mL, compared to the IC_50_ values for the standard Trolox of 393.95 ± 0.19 μg/mL. The free radicals scavenging activities for *A. clematitis* organic ethanolic extract gave a percentage inhibition of 69.58 ± 0.07, almost three times higher than the one obtained by Papuc et al. (2010) [33], which exhibited a DPPH inhibition of 24.77 ± 3.28 [33]. Other studies showed antioxidant activity with the DPPH method on other *Aristolochia* species, for instance *A. baetica* and *A. paucinervis* methanolic extracts, which present IC_50_ values of 150 ± 8 μg/mL and 160 ± 10 μg/mL, respectively, when compared to ascorbic acid [47], and *A. longa* organic extract with an IC_50_ value of 125.40 ± 2.40 μg/mL [48]. *A. clematitis*’s capacity to scavenge DPPH radicals has demonstrated high value, indicating that the extract has low antioxidant activity because it requires a larger amount to achieve the desired level of radical scavenging [49]. Another specie of *Aristolochia*, *A. tagala*, presented antioxidant effects when leaves were extracted in a hydroalcoholic solvent system (30.80 µg/mL) [27]. A study on *A. clematitis* roots methanolic extracts from Algeria showed an IC_50_ of 142 µg/mL [29], and its antioxidant capacity was attributed to the active compounds and the synergy among them.

To the best of our knowledge, no previous data were available for the *A. clematitis* ABTS assay. An IC_50_ for the herbal extract was estimated at 160.89 ± 0.21 μg/mL, and a value of 157.90 ± 0.18 μg/mL was obtained for Trolox, respectively; in other experiments performed on *A. bracteata* ethanolic extracts, an IC_50_ value of 19.42 μg/mL for ABTS scavenging activity was obtained [50].

The total polyphenolic content of *A. clematitis* was quantified after an adapted Folin–Ciocâlteu method [51]. The ethanolic extract of *A. clematitis* has been shown to have a total polyphenolic content of 3.55 ± 0.006 mg GAE/g. One study presents the TPC of an ethanolic extract obtained from rhizomes and the results were 91.3 ± 4.75 mg GAE/g of extract [52]. Another study on phenolic content performed on *A. clematitis* aerial parts extracted in 60% ethanol revealed a 4.6 ± 1.88 mg caffeic acid equivalent/100 mL extract [46]. Additional research showed that the total polyphenolic content of two other species of *Aristolochia* was estimated at 360 ± 20 mg GAE/g and 280 ± 27 mg GAE/g [47]. In another study, the phenolic content determined on *A. talaga* showed a value of 52.96 ± 0.04 mg GAE/g [53], and on a hydroalcoholic and methanolic type of extract, values around 50 mg GAE/g extract were obtained [27]. The low phenol content suggests that other molecules with a different polarity, likely generated by hydrolysis, and other cleavage events may be responsible for the high antioxidant effect that has been experimentally obtained.

The total flavonoid content of *A. clematitis* was estimated at 60.83 ± 0.01 mg QE/g. Also, in another study on *A. clematitis* performed on rhizomes ethanolic extract obtained at 35 °C, the results were five times higher than ours (295.8 ± 12.6 mg QE/g plant) [52]. Also, Crivineanu et al. (2009) used *A. clematitis* aerial parts 60% ethanolic extract and obtained a value of 13.35 ± 3.17 µg QE/mL [46]. The experiment described by Saeed et al. was followed in order to determine the total flavonoid content of *A. clematitis* [54]. Our results were consistent with those described in previous research, where the flavonoid content in *A. tagala* methanolic extract was estimated at 51.65 ± 0.11 mg QE/g [53], and around 50 mg QE/g in hydroalcoholic and methanolic leaf extracts’ TFC [27].

The total antioxidant capacity of plant extracts has been routinely evaluated using the phosphomolybdate method [41]. The TAC of the extracts was evaluated at 4.82 ± 0.03 mg/mL. A higher absorbance value indicates a higher level of antioxidant activity [55]. This method of total antioxidant capacity was performed for the first time for this *Aristolochia* sp. In the current literature, the phosphomolybdenum method is used for aristolochic acids isolated from *A. bracteolata* leafs and stems methanolic extract [42].

The toxicity of *Aristolochia* extracts on Caco-2 cells was investigated. The obtained IC_50_ for both exposure timeframes, 24 h and 48 h, suggest that the extracts are only weakly active on Caco-2 cells at these chosen concentrations. Our results showed cytotoxic effects almost similar for both timeframes and concentrations, 216 µg/mL at 24 h and 224 µg/mL at 48 h, respectively, and closer to Trolox 207 µg/mL.

In different studies, aqueous and organic extracts from *Aristolochiaceae* family plants were evaluated for cytotoxic activity, by MTT or sulforhodamine B (SRB) colorimetric assays, on human cancer cell lines. The difference between cytotoxic activities of *Aristolochia* sp. extracts can be explained by several parameters: the specific particularities of the *Aristolochia* plants; the part of the plant used in the study; the different composition of extracts due to the use of different aqueous or organic solvents; the type of extraction; and the particular features of the cancer cell line. Table 2 encompasses the results of investigations from the literature into the effects of organic extracts based on ethanolic solvents from various species of the genus *Aristolochia* on different cancer cell lines.

The ethanolic extract of the *A. ringens* root was significantly active for all cancer cell lines with IC_50_ values between 3 and 24 µg/mL [19]. *A. galeata* ethanolic extract obtained from rhizomes by maceration for 10 days at room temperature and a hydroalcoholic fraction had the lowest cytotoxicity on the HeLa cell line by the MTT assay after 72 h. Only the organic dichloromethane fraction presented cytotoxic effects [56]. In other experiments, *Aristolochia bracteolata* Lam. leaves were extracted not only with ethanol, but also in combination with other solvents, like methanol and ethyl acetate, and their cytotoxicity was tested by the MTT assay on the Vero cell line and A549 adenocarcinoma cell line. The extract had a cytotoxic effect on cancer cells at a low concentration, while a higher concentration induced cell growth [32]. On the HCT-15 cell line, and its multidrug-resistant subline, HCT-15/CLO2, the extract exhibited potent cytotoxic activities with growth inhibition (GI_50_) values in the micromolar range (>10 µM) [57]. According to current research, the most potent cytotoxic effects of *Aristolochia* sp. come from extracts obtained in different organic solvent systems (<100 µg/mL) [58,59,60,61,62,63].

Furthermore, Trolox showed a higher antioxidant effect on Caco-2 cells compared to the extract (Figure 3). This type of assay was implemented for the first time in the present study. Trolox was used as a control compound because of its use in the calibration curve calculations and because of its use in antioxidant capacity evaluations of *A. clematitis* extracts. This type of cell line was used because of its good reproducibility and predictability of antioxidant activities in vivo [64]. *Aristolochia* sp. extracts and Trolox have in vitro antioxidant activities, and these can be inhibited by low doses of aristolochic acids present in leaves. AAs have cytotoxic effects and their presence in extracts induces ROS production by activating the caspase pathway [65]. *A. debilis* stems methanolic extracts demonstrated dose–time inhibiting effects, on the HT-29 human colon cancer cell line, due to ROS overproduction [66].

This study brings into light new data which are useful especially for alternative medicine approaches, as well as for toxicologists and physicians who diagnose and treat patients with AA-induced kidney injuries. *Aristolochia* sp. extracts can also be used in nanomedicine by synthesizing new types of silver nanoparticles [67,68]. Information about *Aristolochia clematitis* products is available on the website of the Natural Medicines Comprehensive Database [69]. Therefore, data about the safety of medicinal products are presented in the current system of monitoring but the adverse effects are incompletely described. The risks associated with the use of *Aristolochia* sp.-based herbal medicinal products can be reduced by their short-term use as complementary treatment in inflammatory diseases and cancer associated with gastrointestinal tract [3]. Further interdisciplinary collaboration between research scientists and physicians could lead to improved phytotherapeutically formulas by refining extraction protocols and removing the phytotoxins, and guarantee secure and effective integration of these medicinal plants into contemporary therapeutics.

## 4. Materials and Methods

### 4.1. Chemicals and Reagents

Analytical grade ethanol purchased from Sigma-Aldrich (Merck Group, Darmstadt, Germany) was used for extractions and dilutions. For the antioxidant activity assays, the following reagents were used: 6-Hydroxy-2,5,7,8-tetramethylchroman-2-carboxylic acid (Trolox); 1,1-diphenyl-2-picrylhydrazyl radical (DPPH); 2,2′-azino-bis (3-ethylbenzothiazoline-6-sulfonic acid) diammonium salt (ABTS), potassium persulfate (K_2_S_2_O_8_); ammonium molybdate tetrahydrate, sodium phosphate dibasic, sulphuric acid; and Folin–Ciocâlteu reagent, sodium carbonate, propyl gallate; sodium nitrite (NaNO_2_), aluminum chloride hexahydrate(AlCl_3_*6H_2_O), sodium hydroxide (NaOH), all purchased from Sigma-Aldrich (Merck Group, Darmstadt, Germany).

### 4.2. Raw Material Plant Collection and Extraction

*Aristolochia clematitis* L. plants were harvested from Dudeştii Noi, Timiş County (Romania) (45°50′51″ N 21°06′30″ E) in September 2023. A voucher specimen (CBG-AcL-01) has been identified by a botanist and the plant was stored in the Herbarium of the Faculty of Chemistry, Biology, Geography from Timișoara, Romania. The collected specimens were air-dried in the dark at room temperature (RT) and only leaves were ground and used in the extraction process. One gram of leaves was mixed with 15 mL of ethanol and left for maceration under a continuous homogenization at RT for 24 h. *Aristolochia clematitis* ethanolic extract samples (AcE) were centrifuged at 3000 rpm for 5 min and 14 ± 0.3 mL of supernatant was collected, filtered through a 0.45 μm polyethersulfone (PES) filter (Merck Group, Darmstadt, Germany), and kept at 4–8 °C for future analyses.

### 4.3. Antioxidant Activity Evaluation Techniques

#### 4.3.1. The Trolox Equivalent Antioxidant Capacity (TEAC) Assay

For TEAC assays, DPPH, ABTS, and TAC, respectively, were used and 2 mM Trolox was used as a positive standard for calculation. To obtain a calibration curve, dilutions ranging from 0.5 mg/mL to 3 µg/mL were used. As a negative control, we used the specific extraction solvent, ethanol. Also, dilutions of extracts were performed to calculate the inhibition percentage and further, the obtained calibration curve was used to calculate the IC_50_. Therefore, the IC_50_ (μg/mL) value, defined as the concentration of extract required to scavenge the formation of 50% of free radicals, was calculated using Excel software Version 130.0.2849.52 (Microsoft Corporation, Redmond, WA, USA) and expressed as mean ± standard deviation (SD) of three independent experiments. Samples and standards were plated in quadruplicates on 96 round-bottom transparent well plates and their absorbances were registered on a spectrophotometer Tecan i-control, 1.10.4.0 infinite 200Pro. The particularities for each type of TEAC assays are described below.

##### DPPH Radical Scavenging Assay

DPPH radical scavenging assay was conducted using the stable radical, DPPH [39]. A 2.54 mM DPPH methanolic stock solution was used for a calibration curve including concentrations ranging from 0.1 mg/mL to 3.1 μg/mL. Plant extracts diluted 1:10 in the specific solvents were mixed in a ratio of 1:8 (*v*/*v*) with 0.25 mM DPPH methanolic solution and incubated in the dark at room temperature. After a 30 min reaction period, samples were registered at 517 nm. The following equation was used to compute the DPPH scavenging activity:

%DPPH scavenging activity = (A_control_ − A_AcE_) × 100/A_control_,

where A_control_ and A_AcE_ are the absorbances of the control, ethanol, and the extract.

##### ABTS Radical Scavenging Assay

ABTS radical scavenging activity was conducted using an adapted method [63]. ABTS (1 M) and K_2_S_2_O_8_ (2.45 mM) were combined 1:1 (*v*/*v*) in an amber-colored bottle for the preparation of the ABTS cation (ABTS^+^) and kept in the dark at RT for 14 h. The obtained ABTS^+^ solution was diluted with the specific solvents for each type of extract (1:20) to an absorbance of 0.700 ± 0.035 at 734 nm. Subsequently, 300 μL of ABTS^+^ solution was mixed with 100 μL of each extract and left at RT in the dark for 30 min; absorbances were measured at 734 nm. The following equation was used:

%ABTS+• inhibition = (A_control_ − A_AcE_) × 100/A_control_,

where A_control_ measures the absorbance ABTS+• solution mixture without adding the sample, and A_AcE_ measures the absorbance of the sample extract and ABTS+• solution mixture.

##### Total Antioxidant Activity (TAC) Assay

The total antioxidant capacity (TAC) of samples was determined by the method described by Prieto et al., 1999 [41]. The assay is based on the reduction of Mo(VI) to Mo(V) by samples and formation of a green-colored phosphate/Mo(V) complex. A ratio of 1:5 (*v*/*v*) sample to reaction mixture containing 0.6 M sulphuric acid, 28 mM sodium phosphate dibasic and 4 mM ammonium molybdate tetrahydrate was combined in Eppendorf test tubes. The test tubes were incubated at 95 °C for 90 min to complete the reaction, and then cooled at RT to measure absorbances at 695 nm.

#### 4.3.2. Total Phenolic Content Assay (TPC)

The total phenolic content (TPC) of the samples was performed with an adapted Folin–Ciocâlteu method, as previously described with minor modifications [70,71]. Briefly, 1 mL of each extract was mixed in a 1:5 ratio sample with Folin–Ciocâlteu reagent (diluted 1:10 in distilled water to obtain a 0.25 N concentration) and left for five minutes in the dark at room temperature; after this, an equal volume of 7.5% sodium carbonate solution with Folin–Ciocâlteu reagent was added, mixed, and left in the dark at RT for 1 h. The absorbance of the samples was measured at 725 nm. TPC was expressed in gallic acid equivalents (mg GAE/g extract) calculated after a propyl gallate calibration curve with concentrations between 0.15 mg/mL and 1.2 µg/mL.

#### 4.3.3. Total Flavonoid Content Assay (TFC)

The total flavonoid content (TFC) of different extracts was determined by a colorimetric method using aluminum chloride (AlCl_3_) [72]. One mL of each extract was mixed sequentially in 30% methanol with aqueous solutions of 0.3 M AlCl_3_*6H_2_O, 0.5 M NaNO_2_, and 1 M NaOH. The mixtures were kept in the dark at RT for 10 min and after that, the absorbance was measured at 510 nm. The concentration of flavonoids was calculated using a quercetin standard curve (2.5 mg/mL to 78 µg/mL) and expressed as milligrams of quercetin equivalent (QE) per gram of dried leaves (mg QE/g dried leaves).

### 4.4. In Vitro Assays

#### 4.4.1. Caco-2 Cell Cultures

Caco-2 cells were obtained from the American Type Culture Collection (ATCC, Manassas, VA, USA) and expanded in DMEM cell culture media. When the cell culture reached 80% confluence, cells were dispersed with 0.025 M trypsin-EDTA and reseeded in a new flask. The passage number of the cells used in the experiments was between 25 and 39. The culture medium was replaced every 48 h. Cells were cultivated with the specific culture media which included Dulbecco’s modified Eagle’s medium nutrient mixture F-12 HAM (DMEM-F12), supplemented with 20% Fetal Calf Serum and 1% mixture of Penicillin/Streptomycin. All cultures were plated and incubated at 37 °C with 5% CO_2_. Caco-2 cells were left in culture plates to proliferate until a confluence of 90% was reached and were subsequently split into culture plates for further assays.

#### 4.4.2. MTT Assay

*A. clematitis* ethanolic extract (AcE) and Trolox effects on Caco-2 cells were evaluated by the 3-(4,5-dimethylthiazol-2-yl)-2,5-diphenyltetrazolium bromide tetrazolium reduction (MTT) assay (Sigma-Aldrich, Merck Group, Darmstadt, Germany). Briefly, Caco-2 cells, passage 5, and 7 were seeded in 96-well plates (7000 cells/well) and incubated at 37 °C in an atmosphere of 5% CO_2_. For further cytotoxicity assays, an IC_50_ curve was established; therefore, different dilutions of ethanolic extract (from 100 to 600 µg/mL) and Trolox (from 0.125 to 0.5 mg/mL) were tested in four replicates in two independent experiments. Caco-2 cells were used as an absolute control, and ethanol as a negative control. After incubation with the extract and Trolox for 24 and 48 h, 100 µL of MTT solution (5 mg/mL) was added. After 2 h incubation, 100 µL of stop solution was added to inhibit mitochondrial activity, and the absorbances were measured at 570 nm with a reference at 655 nm (spectrophotometer Tecan i-control, 1.10.4.0 infinite 200Pro), according to the manufacturer’s specifications [73]. The absorbance of the treated cultures was subtracted from the absorbance of the untreated cultures and divided by the absorbance of the untreated cultures. The results were multiplied by 100 to calculate the inhibition percent of the viable cells. To calculate the percentage of cytotoxicity, the following equation was used:

% Inhibition = (100 × (Control cells − Sample cells))

Then the inhibition percent was used to obtain a scatter plot against the concentrations of the extracts and the linear curve were used in the calculation of the IC_50_ for the tested AcE and Trolox.

#### 4.4.3. *A. clematitis* Ethanolic Extract and Trolox Effects on Caco-2 Cells by ABTS Method

Ethanolic plant extracts and Trolox effects were tested on Caco-2 cells alone or in combinations after a concentration corresponding to the IC_50_ calculated by the MTT assay was established. Caco-2 cells were cultivated (1 × 10^6^ cells/well) in six-well plates, where compounds were tested in two groups:

- Group 1: cells were exposed to Trolox (2 mM) and ethanolic extract (AcE) (150 µM) for 24 h;
- Group 2: cells were exposed to ethanol for 24 h; also, Caco-2 cells without compounds were used as absolute control.

After exposure of the cells to the compounds, cells were trypsinized, and split for the ABTS experiments. Cells were washed three times in PBS (pH 7.4), centrifuged at 1000 rpm for 10 min, and the supernatant discarded. The pellet was resuspended in 100 µL PBS (pH 7.4), vortexed for homogenization, sonicated on ice, and centrifuged at 10,000 rpm for 15 min at 4 °C. One hundred µL of cell supernatant was mixed with 200 µL ABTS working solution and left to react at RT for 30 min, and then plated on 96 transparent well plates to read the absorbances at 734 nm. Also, a calibration curve with Trolox concentrations ranging between 0.25 mg/mL and 31 µg/mL was made to calculate the concentrations of cell samples, based on the formula below:

T-AOC = (A_734_-b) ÷ a ÷ No. of cells × f

Y—A_control_–A_Trolox_; x—concentration of standard; a—slope of standard curve; b—intercept of standard curve; A_734_—A_control_–A_AcE_; f—dilution factor of sample before test; and no. of cells—number of Caco-2 cells in sample per mL of PBS.

The results for the tested Trolox and AcE concentrations were generated from four replicates from two independent experiments.

### 4.5. Statistical Analysis

The statistical analyses were performed using the Microsoft Office-Excel 2010 Data Analysis Tool (Microsoft Corporation, Redmond, WA, USA) package. The results were calculated and presented as average ± standard deviation (SD), and significant differences were mentioned (*p* < 0.05).

## 5. Conclusions

The study carried out on the *A. clematitis* L. leaf extracts obtained by maceration in ethanol at room temperature, a formulation similar to the traditional one used in ethnomedicine, revealed for the first time the antioxidant activities of such extracts, which are correlated with their cytotoxic effects on a human colon cancer cell line. Antioxidant activity values obtained from the three types of chemical-based assays calculated with Trolox showed a weak response of the extract (>200 µg/mL) by DPPH and TAC, but moderate response (<150 µg/mL) by the ABTS assay. The total phenolic content showed a low antioxidant activity based on polyphenols, but a moderate activity on total flavonoid content based on a high level in flavonoids. To our knowledge, these results are obtained for the first time for this type of extract and they demonstrat a moderate antioxidant activity with the cell-based ABTS assay. Also, the ABTS assay used to test the antioxidant activity in the tested cells showed similar effects between the extracts and Trolox. The IC_50_ values obtained by the MTT cytotoxicity assay represent a weak response of the extract on the Caco-2 cells at two timeframes. Therefore, the extract in this formulation used in traditional medicine could be cytotoxic at higher concentrations and after a longer period of time. *A. clematitis* extracts may represent a curative option and a source of low-cost antioxidants, provided short-term use and proper dosage, which could be included into modern healthcare practices. These chemical-based antioxidant assays are low cost and easy to perform and can be used as screening methods of natural products by the scientific or medical community, prior to their chemical characterization.

## Figures and Tables

**Table 1:** The antioxidant activities of *A. clematitis* ethanolic extract tested with five different assays.

Samples	TEAC IC_50_ (µg/mL)			TPC (mg GAE/g DW)	TFC (mg Q/g DW)
	DPPH	ABTS	TAC		
AcE	392.62 ± 0.06	160.89 ± 0.21	4816.37 ± 0.03	3.55 ± 0.006	60.83 ± 0.01
Trolox	393.95 ± 0.19	157.90 ± 0.18	4271.28 ± 0.002	-	-

**Table 2:** The IC_50_ values of organic extracts including alcoholic extracts of the *Aristolochiaceae* family.

Cell Line	Species	Part of the Plant	Type of Extract	Type of Solvent	IC_50_ (µg/mL)	Ref
A549 (lung) HCT-116 (colon) PC3 (prostate) A431 (skin) HeLa (cervix) THP-1 (leukemia)	*A. ringens* Vahl.	root	macerated	EtOH/HydroEtOH/EtOH + HydroEtOH/DCM-MeOH	20/ > 100/ > 100/26 22/ > 100/ > 100/19.5 3/ > 100/ > 100/12 -/ > 100/ > 100/28 -/ > 100/ > 100/30 24/ > 100/ > 100/22	[19]
HeLa	*A. galeata* Mart.	rhizomes	macerated	EtOH/hexane/DCM/ethyl acetate/hydroEtOH	369/726/90/1620/1340	[56]

EtOH—ethanol; DCM—dichloromethane; and MeOH—methanol.

## Data Availability

The data presented in this study are available on request from the corresponding authors.

## References

[1] Ang L.P., Ng P.W., Lean Y.L., Kotra V., Kifli N., Goh H.P., Lee K.S., Sarker M.M.R., Al-Worafi Y.M., Ming L.C. (2021). Herbal Products Containing Aristolochic Acids: A Call to Revisit the Context of Safety. J. Herb. Med..

[2] Michl J., Jennings H.M., Kite G.C., Ingrouille M.J., Simmonds M.S.J., Heinrich M. (2013). Is Aristolochic Acid Nephropathy a Widespread Problem in Developing Countries?. J. Ethnopharmacol..

[3] Kiliś-Pstrusińska K., Wiela-Hojeńska A. (2021). Nephrotoxicity of Herbal Products in Europe—A Review of an Underestimated Problem. Int. J. Mol. Sci..

[4] Scarborough J. (2011). Ancient Medicinal Use of *Aristolochia*: Birthwort’s Tradition and Toxicity. Pharm. Hist..

[5] Kuo P.-C., Li Y.-C., Wu T.-S. (2012). Chemical Constituents and Pharmacology of the *Aristolochia* (馬兜鈴 Mădōu Ling) Species. J. Tradit. Complement. Med..

[6] Bartha G.S., Tóth G., Horváth P., Kiss E., Papp N., Kerényi M. (2020). Analysis of Aristolochlic Acids and Evaluation of Antibacterial Activity of *Aristolochia clematitis* L. BioFut.

[7] Heinrich M., Chan J., Wanke S., Neinhuis C., Simmonds M.S.J. (2009). Local Uses of *Aristolochia* Species and Content of Nephrotoxic Aristolochic Acid 1 and 2—A Global Assessment Based on Bibliographic Sources. J. Ethnopharmacol..

[8] Michl J., Ingrouille M.J., Simmonds M.S.J., Heinrich M. (2014). Naturally Occurring Aristolochic Acid Analogues and Their Toxicities. Nat. Prod. Rep..

[9] Chen C.H., Dickman K.G., Huang C.Y., Moriya M., Shun C.T., Tai H.C., Huang K.H., Wang S.M., Lee Y.J., Grollman A.P. (2013). Aristolochic Acid-Induced Upper Tract Urothelial Carcinoma in Taiwan: Clinical Characteristics and Outcomes. Int. J. Cancer.

[10] Dickman K.G., Chen C.-H., Grollman A.P., Pu Y.-S. (2022). Aristolochic Acid-Containing Chinese Herbal Medicine and Upper Urinary Tract Urothelial Carcinoma in Taiwan: A Narrative Review. World J. Urol..

[11] Chen I.-H., Luo H.-L., Su Y.-L., Huang C.-C., Chiang P.-H., Yu C.-C., Lee N.-L., Lin J.-J., Sung M.-T. (2019). Aristolochic Acid Affects Upper Tract Urothelial Cancer Behavior through the MAPK Pathway. Molecules.

[12] Nortier J.L. (2003). Invasive Urothelial Carcinoma after Exposure to Chinese Herbal Medicine Containing Aristolochic Acid May Occur without Severe Renal Failure. Nephrol. Dial. Transplant..

[13] Jadot I., Declèves A.-E., Nortier J., Caron N. (2017). An Integrated View of Aristolochic Acid Nephropathy: Update of the Literature. Int. J. Mol. Sci..

[14] Arlt V.M., Stiborova M., Vom Brocke J., Simoes M.L., Lord G.M., Nortier J.L., Hollstein M., Phillips D.H., Schmeiser H.H. (2007). Aristolochic Acid Mutagenesis: Molecular Clues to the Aetiology of Balkan Endemic Nephropathy-Associated Urothelial Cancer. Carcinogenesis.

[15] Anger E.E., Yu F., Li J. (2020). Aristolochic Acid-Induced Nephrotoxicity: Molecular Mechanisms and Potential Protective Approaches. Int. J. Mol. Sci..

[16] Arlt V.M., Ferluga D., Stiborova M., Pfohl-Leszkowicz A., Vukelic M., Ceovic S., Schmeiser H.H., Cosyns J.-P. (2002). Is Aristolochic Acid a Risk Factor for Balkan Endemic Nephropathy-Associated Urothelial Cancer?. Int. J. Cancer.

[17] International Agency for Research on Cancer (2012). IARC Monographs on the Evaluation of Carcinogenic Risks to Humans, Volume 100 D, Radiation: This Publication Represents the Views and Expert Opinions of an IARC Working Group on the Evaluation of Carcinogenic Risks to Humans, Which Met in Lyon, 2–9 June 2009.

[18] Grollman A.P., Marcus D.M. (2016). Global Hazards of Herbal Remedies: Lessons from *Aristolochia*: The Lesson from the Health Hazards of *Aristolochia* Should Lead to More Research into the Safety and Efficacy of Medicinal Plants. EMBO Rep..

[19] Akindele A.J., Wani Z., Mahajan G., Sharma S., Aigbe F.R., Satti N., Adeyemi O.O., Mondhe D.M. (2015). Anticancer Activity of *Aristolochia ringens* Vahl. (*Aristolochiaceae*). J. Tradit. Complement. Med..

[20] Alok S., Jain S.K., Verma A., Kumar M., Mahor A., Sabharwal M. (2014). Herbal Antioxidant in Clinical Practice: A Review. Asian Pac. J. Trop. Biomed..

[21] Sun J., Pan J., Liu Q., Cheng J., Tang Q., Ji Y., Cheng K., Wang R., Liu L., Wang D. (2023). Melatonin Attenuates Mitochondrial Damage in Aristolochic Acid-Induced Acute Kidney Injury. Biomol. Ther..

[22] Efferth T., Oesch F. (2021). Repurposing of Plant Alkaloids for Cancer Therapy: Pharmacology and Toxicology. Semin. Cancer Biol..

[23] Lerma-Herrera M.A., Beiza-Granados L., Ochoa-Zarzosa A., López-Meza J.E., Navarro-Santos P., Herrera-Bucio R., Aviña-Verduzco J., García-Gutiérrez H.A. (2022). Biological Activities of Organic Extracts of the Genus *Aristolochia*: A Review from 2005 to 2021. Molecules.

[24] Košťálová D., Hrochová V., Pronayová N., Leško J. (1991). Constituents of *Aristolochia clematitis* L. Chem. Papers.

[25] Wu T.-S., Chan Y.-Y., Leu Y.-L., Chen Z.-T. (1999). Sesquiterpene Esters of Aristolochic Acid from the Root and Stem of *Aristolochia Heterophylla*. J. Nat. Prod..

[26] Wu T.-S., Damu A.G., Su C.-R., Kuo P.-C. (2004). Terpenoids of *Aristolochia* and Their Biological Activities. Nat. Prod. Rep..

[27] Mariyammal V., Sathiageetha V., Amalraj S., Gurav S.S., Amiri-Ardekani E., Jeeva S., Ayyanar M. (2023). Chemical Profiling of *Aristolochia tagala* Cham. Leaf Extracts by GC-MS Analysis and Evaluation of Its Antibacterial Activity. J. Indian Chem. Soc..

[28] Podea R., Culea M., Fromondi L. (2001). The Determination of the Therapeutic Compounds from Aristolochia clematitis by GC/MS.

[29] Benmehdi H., Behilil A., Memmou F., Amrouche A. (2017). Free Radical Scavenging Activity, Kinetic Behaviour and Phytochemical Constituents of *Aristolochia clematitis* L. Roots. Arab. J. Chem..

[30] Egbujor M.C., Egu S.A., Okonkwo V.I., Jacob A.D., Egwuatu P.I., Amasiatu I.S. (2021). Antioxidant Drug Design: Historical and Recent Developments. J. Pharm. Res. Int..

[31] Xu D.-P., Li Y., Meng X., Zhou T., Zhou Y., Zheng J., Zhang J.-J., Li H.-B. (2017). Natural Antioxidants in Foods and Medicinal Plants: Extraction, Assessment and Resources. Int. J. Mol. Sci..

[32] Sreeharsha N., Kathiravan G., Rajagopal K., Rajasekar A., Rajangam B., Aldhubiab B., Narayanaswamy V., Attimarad M., Nair A., Karnathi R. (2020). Synthesis, Characterization, and Biological Activity of Silver Nanoparticles Synthesized from *Aristolochia bracteolata* Lam. Pharmacogn. Mag..

[33] Papuc C., Crivineanu M., Goran G., Nicorescu V., Durdun N. (2010). Free Radicals Scavenging and Antioxidant Activity of European Mistletoe (*Viscum album*) and European Birthwort (*Aristolochia clematitis*). Rev. Chim..

[34] Barba-Ostria C., Carrera-Pacheco S.E., Gonzalez-Pastor R., Heredia-Moya J., Mayorga-Ramos A., Rodríguez-Pólit C., Zúñiga-Miranda J., Arias-Almeida B., Guamán L.P. (2022). Evaluation of Biological Activity of Natural Compounds: Current Trends and Methods. Molecules.

[35] Mendonça J.D.S., Guimarães R.D.C.A., Zorgetto-Pinheiro V.A., Fernandes C.D.P., Marcelino G., Bogo D., Freitas K.D.C., Hiane P.A., De Pádua Melo E.S., Vilela M.L.B. (2022). Natural Antioxidant Evaluation: A Review of Detection Methods. Molecules.

[36] Christodoulakis N.S., Kotsironi K., Tsafantakis N., Stefi A.L., Fokialakis N. (2019). Leaf Structure and Phytochemical Analysis of *Aristolochia baetica*, a Traditionally Used Pharmaceutical Plant. J. Herbs Spices Med. Plants.

[37] Kimura O., Haraguchi K., Ohta C., Koga N., Kato Y., Endo T. (2014). Uptake of Aristolochic Acid I into Caco-2 Cells by Monocarboxylic Acid Transporters. Biol. Pharm. Bull..

[38] Aiello P., Sharghi M., Mansourkhani S.M., Ardekan A.P., Jouybari L., Daraei N., Peiro K., Mohamadian S., Rezaei M., Heidari M. (2019). Medicinal Plants in the Prevention and Treatment of Colon Cancer. Oxidative Med. Cell. Longev..

[39] Brand-Williams W., Cuvelier M.E., Berset C. (1995). Use of a Free Radical Method to Evaluate Antioxidant Activity. LWT—Food Sci. Technol..

[40] Ozgen M., Reese R.N., Tulio A.Z., Scheerens J.C., Miller A.R. (2006). Modified 2,2-Azino-Bis-3-Ethylbenzothiazoline-6-Sulfonic Acid (ABTS) Method to Measure Antioxidant Capacity of Selected Small Fruits and Comparison to Ferric Reducing Antioxidant Power (FRAP) and 2,2‘-Diphenyl-1-Picrylhydrazyl (DPPH) Methods. J. Agric. Food Chem..

[41] Prieto P., Pineda M., Aguilar M. (1999). Spectrophotometric Quantitation of Antioxidant Capacity through the Formation of a Phosphomolybdenum Complex: Specific Application to the Determination of Vitamin E. Anal. Biochem..

[42] Al-Busafi S., Al-Harthi M., Al-Sabahi B. (2004). Isolation of Aristolochic Acids from *Aristolochia bracteolata* and Studies of Their Antioxidant Activities. SQU J. Sci..

[43] Phongpaichit S., Nikom J., Rungjindamai N., Sakayaroj J., Hutadilok-Towatana N., Rukachaisirikul V., Kirtikara K. (2007). Biological Activities of Extracts from Endophytic Fungi Isolated from *Garcinia* Plants: Biological Activities of Extracts from Endophytic Fungi. FEMS Immunol. Med. Microbiol..

[44] Gyurászová M., Gurecká R., Bábíčková J., Tóthová Ľ. (2020). Oxidative Stress in the Pathophysiology of Kidney Disease: Implications for Noninvasive Monitoring and Identification of Biomarkers. Oxidative Med. Cell. Longev..

[45] López-Alarcón C., Denicola A. (2013). Evaluating the Antioxidant Capacity of Natural Products: A Review on Chemical and Cellular-Based Assays. Anal. Chim. Acta.

[46] Crivineanu M., Durdun C., Nicorescu I. (2009). Antioxidant Activity of Some Polyphenolic Extracts Obtained from Plants with Antitumoral Potential on Linoleic Acid Emulsion. Bull. Univ. Agric. Sci. Vet. Med. Cluj-Napoca Vet. Med..

[47] Bourhia M., Laasri F.E., Moussa S.I., Ullah R., Bari A., Saeed Ali S., Kaoutar A., Haj Said A.A., El Mzibri M., Said G. (2019). Phytochemistry, Antioxidant Activity, Antiproliferative Effect, and Acute Toxicity Testing of Two Moroccan *Aristolochia* Species. Evid.-Based Complement. Altern. Med..

[48] El Omari N., Sayah K., Fettach S., El Blidi O., Bouyahya A., Faouzi M.E.A., Kamal R., Barkiyou M. (2019). Evaluation of In Vitro Antioxidant and Antidiabetic Activities of *Aristolochia longa* Extracts. Evid.-Based Complement. Altern. Med..

[49] Olugbami J.O., Gbadegesin M.A., Odunola O.A. (2014). In Vitro Evaluation of the Antioxidant Potential, Phenolic and Flavonoid Contents of the Stem Bark Ethanol Extract of *Anogeissus leiocarpus*. Afr. J. Med. Med. Sci..

[50] Jegadeeswari P., Daffodil E.D., Mohan V.R. (2014). Quantification of Total Phenolics, Flavonoid and in Vitro Antioxidant Activity of *Aristolochia bracteata* Retz. Int. J. Pharm. Pharm. Sci..

[51] Jianu C., Goleț I., Stoin D., Cocan I., Lukinich-Gruia A.T. (2020). Antioxidant Activity of *Pastinaca sativa* L. Ssp. Sylvestris [Mill.] Rouy and Camus Essential Oil. Molecules.

[52] Abbouyi A.E., Soukaina E.M., Filali-Ansari N., Khyari S.E. (2016). Antioxidant effect of extract of rhizomes from Aristolochia clematitis. J. Chem. Biol. Phys. Sci. (JCBPS).

[53] Hadem K.H., Sharan R., Kma L. (2016). Phytochemicals of *Aristolochia tagala* and *Curcuma caesia* Exert Anticancer Effect by Tumor Necrosis Factor-α-Mediated Decrease in Nuclear Factor kappaB Binding Activity. J. Basic Clin. Pharma.

[54] Saeed N., Khan M.R., Shabbir M. (2012). Antioxidant Activity, Total Phenolic and Total Flavonoid Contents of Whole Plant Extracts *Torilis leptophylla* L. BMC Complement. Altern. Med..

[55] Nagendra Prasad K., Yang B., Yang S., Chen Y., Zhao M., Ashraf M., Jiang Y. (2009). Identification of Phenolic Compounds and Appraisal of Antioxidant and Antityrosinase Activities from Litchi (*Litchi sinensis* Sonn.) Seeds. Food Chem..

[56] Aleixo Á.A., Camargos V.N., Santos A.C., Andrade P., Marjorie K., Herrera S., Iara R., Ribeiro M., Magalhães J.T., Magalhães J. (2014). Antibacterial and Cytotoxic Antibacterial Potential of Ethanol Extract and Fractions from *Aristolochia galeata* Mart. Ex Zucc. J. Med. Plants Res..

[57] Choi Y.L., Kim J.K., Choi S.-U., Min Y.-K., Bae M.-A., Kim B.T., Heo J.-N. (2009). Synthesis of Aristolactam Analogues and Evaluation of Their Antitumor Activity. Bioorganic Med. Chem. Lett..

[58] Truong L.H., Cuong N.H., Dang T.H., Hanh N.T.M., Thi V.L., Tran Thi Hong H., Quang T.H., Nguyen H.D., Nguyen Xuan C., Nguyen Hoai N. (2021). Cytotoxic Constituents from *Isotrema tadungense*. J. Asian Nat. Prod. Res..

[59] Mongelli E., Pampuro S., Coussio J., Salomon H., Ciccia G. (2000). Cytotoxic and DNA Interaction Activities of Extracts from Medicinal Plants Used in Argentina. J. Ethnopharmacol..

[60] Yu J.Q., Liao Z.X., Cai X.Q., Lei J.C., Zou G.L. (2007). Composition, Antimicrobial Activity and Cytotoxicity of Essential Oils from *Aristolochia mollissima*. Environ. Toxicol. Pharmacol..

[61] Ruffa M.J., Ferraro G., Wagner M.L., Calcagno M.L., Campos R.H., Cavallaro L. (2002). Cytotoxic Effect of Argentine Medicinal Plant Extracts on Human Hepatocellular Carcinoma Cell Line. J. Ethnopharmacol..

[62] Benarba B., Aoues A., Vazquez A., Ambroise G., Meddah B. (2012). *Aristolochia longa* Aqueous Extract Triggers the Mitochondrial Pathway of Apoptosis in BL41 Burkitt′s Lymphoma Cells. Int. J. Green Pharm..

[63] Lerma-Herrera M.A., Beiza-Granados L., Ochoa-Zarzosa A., López-Meza J.E., Hernández-Hernández J.D., Aviña-Verduzco J., García-Gutiérrez H.A. (2021). In Vitro Cytotoxic Potential of Extracts from *Aristolochia* Foetida Kunth against MCF-7 and bMECs Cell Lines. Saudi J. Biol. Sci..

[64] Wan H., Liu D., Yu X., Sun H., Li Y. (2015). A Caco-2 Cell-Based Quantitative Antioxidant Activity Assay for Antioxidants. Food Chem..

[65] Wu T.-K., Wei C.-W., Pan Y.-R., Cherng S.-H., Chang W.-J., Wang H.-F., Yu Y.-L. (2015). Vitamin C Attenuates the Toxic Effect of Aristolochic Acid on Renal Tubular Cells via Decreasing Oxidative Stress-Mediated Cell Death Pathways. Mol. Med. Rep..

[66] Li C., Wang M.-H. (2013). *Aristolochia debilis* Sieb. et Zucc. Induces Apoptosis and Reactive Oxygen Species in the HT-29 Human Colon Cancer Cell Line. Cancer Biother. Radiopharm..

[67] Siva C., Iyappan M., Kumar M.S., Mary R.R. (2011). Green Synthesis of Silver Nanoparticles by Using *Aristolochia indica* Leaf Extract. Indian J. Appl. Res..

[68] Thirumagal D.J. (2018). A Study on Silver Nano Particle Production from *Aristolochia bracteata* and Its Antimicrobial Activity. Int. J. Adv. Res. Dev..

[69] NatMed Pro—Search Results. https://naturalmedicines.therapeuticresearch.com/search.aspx?q=aristolochia+clematitis&go.x=0&go.y=0.

[70] Swain T., Hillis W.E. (1959). The Phenolic Constituents of *Prunus domestica*. I.—The Quantitative Analysis of Phenolic Constituents. J. Sci. Food Agric..

[71] Sánchez-Rangel J.C., Benavides J., Heredia J.B., Cisneros-Zevallos L., Jacobo-Velázquez D.A. (2013). The Folin–Ciocalteu Assay Revisited: Improvement of Its Specificity for Total Phenolic Content Determination. Anal. Methods.

[72] Shraim A.M., Ahmed T.A., Rahman M.M., Hijji Y.M. (2021). Determination of Total Flavonoid Content by Aluminum Chloride Assay: A Critical Evaluation. LWT.

[73] Mosmann T. (1983). Rapid Colorimetric Assay for Cellular Growth and Survival: Application to Proliferation and Cytotoxicity Assays. J. Immunol. Methods.

